# Retinal Vessel Plexus Differentiation Based on OCT Angiography Using Deep Learning

**DOI:** 10.1016/j.xops.2024.100605

**Published:** 2024-08-23

**Authors:** Jamie L. Shaffer, Luis De Sisternes, Anand E. Rajesh, Marian S. Blazes, Yuka Kihara, Cecilia S. Lee, Warren H. Lewis, Roger A. Goldberg, Niranchana Manivannan, Aaron Y. Lee

**Affiliations:** 1 Department of Ophthalmology, UW Medicine, Seattle, Washington; 2 The Roger and Angie Karalis Johnson Retina Center, Seattle, Washington; 3 Research and Development, Carl Zeiss Meditec Inc, Dublin, California; 4 Bayside Photonics, Inc, Yellow Springs, Ohio; 5 Bay Area Retina Associates, Walnut Creek, California

**Keywords:** Deep learning, OCT angiography, Retinal vascular plexus, Retinal vasculature

## Abstract

**Purpose:**

Although structural OCT is traditionally used to differentiate the vascular plexus layers in OCT angiography (OCTA), the vascular plexuses do not always obey the retinal laminations. We sought to segment the superficial, deep, and avascular plexuses from OCTA images using deep learning without structural OCT image input or segmentation boundaries.

**Design:**

Cross-sectional study.

**Subjects:**

The study included 235 OCTA cubes from 33 patients for training and testing of the model.

**Methods:**

From each OCTA cube, 3 weakly labeled images representing the superficial, deep, and avascular plexuses were obtained for a total of 705 starting images. Images were augmented with standard intensity and geometric transforms, and regions from adjacent plexuses were programmatically combined to create synthetic 2-class images for each OCTA cube. Images were partitioned on a per patient basis into training, validation, and reserved test groups to train and evaluate a U-Net based machine learning model. To investigate the generalization of the model, we applied the model to multiclass thin slabs from OCTA volumes and qualitatively observed the resulting b-scans.

**Main Outcome Measures:**

Plexus segmentation performance was assessed quantitatively using Dice scores on a held-out test set.

**Results:**

After training on single-class plexus images, our model achieved good results (Dice scores > 0.82) and was further improved when using the synthetic 2-class images (Dice >0.95). Although not trained on more complex multiclass slabs, the model performed plexus labeling on slab data, which indicates that the use of only OCTA data shows promise for segmenting the superficial, deep, and avascular plexuses without requiring OCT layer segmentations, and the use of synthetic 2-class images makes a significant performance improvement.

**Conclusions:**

This study presents the use of OCTA data alone to segment the superficial, deep, and avascular plexuses of the retina, confirming that use of structural OCT layer segmentations as boundaries is not required.

**Financial Disclosure(s):**

Proprietary or commercial disclosure may be found in the Footnotes and Disclosures at the end of this article.

The retina receives oxygen and nutrients via its blood supply through capillary networks called plexuses that travel through layers of the retina.^1^ Any retinal disease that affects the vasculature may affect the retinal vascular plexuses in a disease dependent manner. Glaucoma, diabetes, hypertension, retinal vascular occlusion, age-related macular degeneration, retinitis pigmentosa, and many other diseases have been associated with varying changes in the plexus layer structure and/or thickness.^2^, ^3^, ^4^, ^5^, ^6^, ^7^ Although it is understood that there are microvascular changes in the capillary network that occur in many ophthalmic diseases, it is not fully understood what these changes are with respect to the different plexus layers. Early studies of the retina identified 2 separate vascular plexuses within the retina: the superficial and deep plexuses.^8^^,^^9^ Although some authors have further subdivided the superficial and deep plexuses into as many as 4 separate vascular plexuses,^10^, ^11^, ^12^, ^13^ these newer divisions still preserve the early distinction between the superficial and deep plexuses.^12^ Posterior to the deep plexus lie the avascular outer layers of the retina, where the lack of vasculature is thought to help reduce any disruption in the light sensing photoreceptors that are located in this area.^14^

Structural OCT can provide detailed information about the various layers of the retina, but the boundaries of vascular plexuses do not always follow the retinal layers that are seen on structural OCT images.^13^ This is because blood vessels are often located within the layers of the retina, and their course may not always correspond directly to the boundaries seen on OCT images. Alternative imaging techniques such as OCT angiography (OCTA) are used to visualize the vasculature without the need for contrast agents. OCT angiography provides detailed images of the retinal microvasculature, allowing for a better understanding of the vascular structure and its relationship to the retinal layers. To assist in further study of retinal plexuses, previous studies have attempted to algorithmically segment the superficial and deep plexuses. These algorithms mostly rely on creating retinal layer segmentation masks from structural OCT images. With the retinal layers segmented on structural OCT automatically or with human correction, the algorithms then infer the plexus boundaries on corresponding OCTA images from the same eye.^15^^,^^16^

This work explores the use of a trained model to operate on OCTA data alone, removing any requirement to comply with the structural layer segmentations commonly used as computational boundaries. Although this approach does not lend itself to direct numerical comparison with OCT structurally constrained approaches, applying the model to a full cube of OCTA data and then reconstructing b-scans provides an opportunity to consider the advantages and limitations of this approach.

Here we demonstrate a fully automated segmentation method for identifying the superficial plexus, deep plexus and avascular outer layer of the retina using only OCTA as input. This has the potential to automatically quantify the retinal vasculature without human intervention or structural OCT to study microvascular changes in ophthalmologic disease.

## Methods

### Dataset Construction

Vascular data from slabs of 235 OCTA cubes (33 patients) consisting of 6 × 6-mm (500 × 500-pixel) en face images × 1536 slices were obtained using a PLEX Elite 9000 (ZEISS) in association with Bay Area Retina Associates. The data included healthy (14 patients) and diseased eyes (19 patients). Diseased eyes included the following pathologic conditions, with some eyes having multiple conditions: wet and dry age-related macular degeneration, diabetic macular edema, epiretinal membrane, intraretinal fluid, subretinal fluid, branch retinal vein occlusion, diabetic retinopathy, posterior vitreous detachment, pigment epithelial detachment, hypertensive related vascular attenuation, and drusen. This study protocol was approved by an Institutional Review Board and was conducted in accordance with the Declaration of Helsinki and compliant with the Health Insurance Portability and Accountability Act of 1996. All participants provided written informed consent.

**Figure 1:**
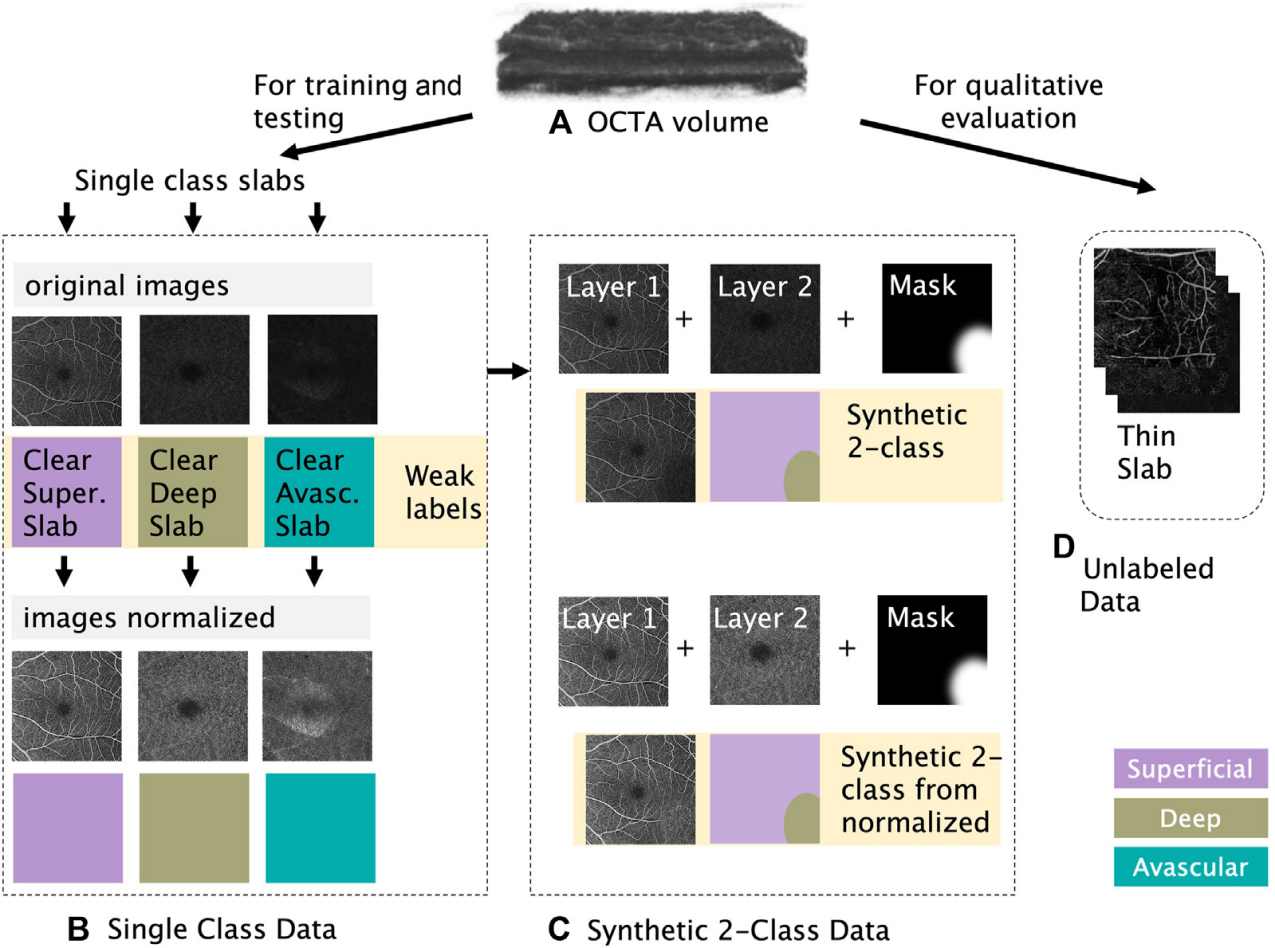
Construction of training and evaluation datasets. **(A)** An OCT angiography volume was preprocessed to create (**B**) single class slabs containing data from only the superficial (Super.) plexus, deep plexus, or avascular (Avasc.) region, along with a normalized version of each image. These single-class images were paired to create (**C**) synthetic 2-class images and their corresponding prediction masks (note that only the case of a deep region inserted into a superficial image is shown). Unlabeled thin slabs (**D**) were created for qualitative analysis from OCTA volumes in the reserved test set. OCTA = OCT angiography.

The OCTA data cubes (Fig 1A) were processed to create several distinct datasets, including single-class images, synthetic 2-class images, and unlabeled thin slabs as described next. Single-class processed images provide clear training examples, 2-class images provide examples of either superficial/deep or deep/avascular that more closely resemble OCTA cube data, and unlabeled thin slabs provide a full cube for qualitative analysis.

#### Single-Class Dataset Construction

**Figure 2:**
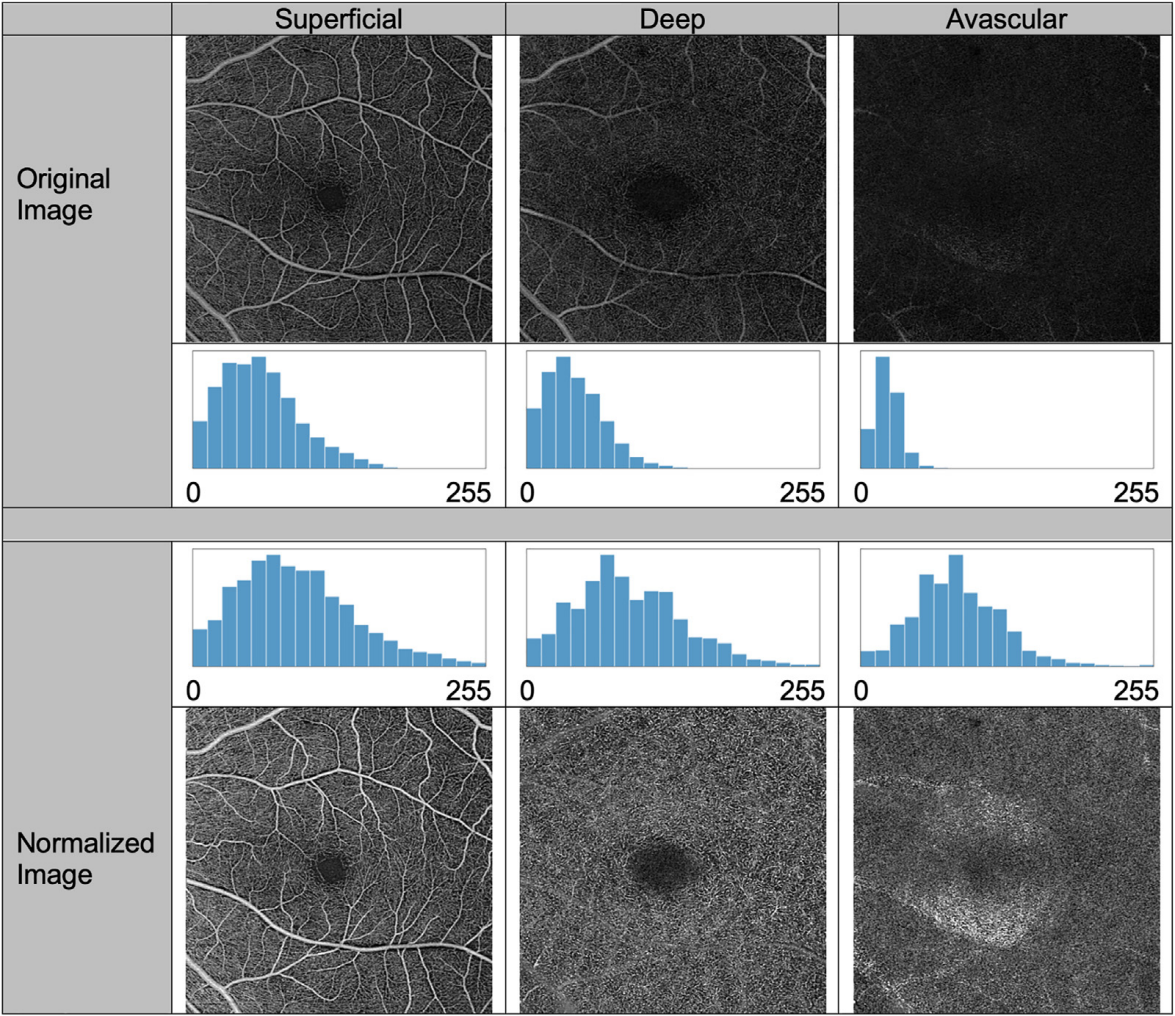
Examples of images before and after normalization in the single class dataset. The percentages of high and low pixel values to adjust were determined empirically so as to create similar pixel value histograms for all 3 classes.

The single-class dataset consists of 235 superficial, 235 deep, and 235 avascular en face OCTA slab projections (1 set from each of the 235 cubes) and their corresponding weakly labeled single-class masks (Fig 1B). The layer segmentation algorithm integrated into the instrument automatically extracted the upper and lower boundaries of every anatomic layer (including superficial, deep, and avascular layers) from the structural OCT volume. In cases where the segmentation contains errors, human reviewers experienced in retinal segmentation (LdS) may observe and then opt to modify the segmentations; in this dataset, no manual editing was performed. The OCTA en face images were projected within each layer's boundaries on the OCTA volume by calculating the maximum values in depth. A slab-based projection removal algorithm was applied to remove the projection artifact on deeper layers. We consider this type of data to be weakly labeled. In this case, individual vessel or plexus regions have not been explicitly annotated by hand to annotate individual vessel or plexus regions, but instead, image-processing techniques and data assumptions were primarily utilized. All pixels in the single-class image were labeled only with the layer segmentation (superficial, deep, or avascular). Structural OCT images, which were captured simultaneously with the OCTA, were utilized as reference images for anatomic layer segmentation and the corresponding manual corrections for the single-class training images. Additionally, they were used as a reference for the qualitative analysis of the model’s predicted class labels. No structural OCT images were used for training or quantitative evaluation. We included original single-class images without modification as well as a second set of the same images that were normalized as follows: adjust the bottom 2% of pixel values to 0, adjust the top 1% of pixel values to 255, and linearly rescale the central 97% of pixel values to span the range of 0 to 255 (Fig 2). The normalized images were intended to adjust the distribution of pixel values for each layer such that the model would focus on the patterns of the images and not rely on the distribution to determine the label; without normalization, the superficial plexus tends to include many bright pixels, and the avascular layer tends to have very few bright pixels. The percentages of high and low values to adjust were determined empirically so as to create similar pixel value histograms for all 3 classes.

#### Synthetic 2-Class Dataset Construction

Leveraging the single-class dataset, we created a dataset of synthetic 2-class images by blending a randomly placed and sized region from an adjacent class into the original image (Fig 1C). We thus created 4 additional images for each of the 235 cubes: blended a small superficial region into a deep image, blended a small deep region into a superficial image, blended a small deep region into an avascular image, and blended a small avascular region into a deep image. For each 2-class pairing, a new grayscale mask was generated to contain an ovoid region with blurred edges to create a blended transition. Blurred ovoid insets were chosen to better represent retinal pathologies and to train a model that might predict complex class transitions rather than learning to expect only sharp or rectangular transitions. The ovoid was constrained to ensure that a full transition from 100% majority class to a much smaller region of 100% minority class occurred over the region of blended edges. This blending mask was then applied to the paired images to create the dataset image; a 50% threshold was applied to the blending mask to create a binary segmentation target. We repeated this process using the normalized versions of the single class images, resulting in a total of 8 additional images for each of the 235 cubes.

#### Thin Slab Dataset Construction

Thin slabs of unlabeled data (no ground truth) were also created (Fig 1D) for use in qualitative evaluation of a trained model. After applying a volume-based projection removal, we created 2-dimensional thin slab images by averaging 5 axial pixel sections (corresponding to approximately a 10-μm vertical slice of the volume) of data through the retinal area of the OCTA volume. The retinal area was defined with structural borders of the internal limiting membrane to retinal pigment epithelium, and the slab contours matched internal limiting membrane and retinal pigment epithelium at the extrema and gradually adjusted from 1 contour to the other through the subvolume, ensuring that all pixels belong to one of the 3 plexuses and not to an untrained class such as the vitreous region. No attempt was made to locate or conform with inner plexiform layer and inner nuclear layer junction or outer plexiform layer and outer nuclear layer junction boundaries in this subvolume.

Original images (500 × 500 pixels) in each dataset were converted to 256 × 256 for training and inference and upscaled to 500 × 500 with nearest-neighbor interpolation for visual analysis.

### Dataset Splits for Training, Validation, and Test

Images in this dataset included both healthy and diseased eyes. Manual partitioning without viewing of images was performed, ensuring that each of the 33 patients was represented in only 1 partition (patient level partitioning), patients with very few image cubes were distributed among the partitions, patients with a large number of image cubes were also distributed among the partitions, and both left and right eyes were represented in each partition. The result was 6 groups: 4 groups ensembled for training (9 healthy, 12 diseased), 1 group for validation (3 healthy, 3 diseased), and 1 reserved test group (2 healthy, 4 diseased) (Table 1).

**Table 1:** Training, Validation, and Reserved Test Groups After Splitting by Patient

	Training Group	Validation Group	Reserved Test Group	Total
Number of patients	21	6	6	33
OD/OS	90/63	28/14	20/20	138/97
Number of cubes	153 (65%)	42 (18%)	40 (17%)	235

OD = right eye (oculus dextrus); OS = left eye (oculus sinister).

### Model Architecture and Training

The plexus labeling task that this work addresses falls into the category of semantic segmentation where there have been many successful architectures, including UNet^17^ and LinkNet.^18^ Inside these models, the structure of the encoder and decoder convolutional layer stacks is termed the backbone of the architecture, and backbones are frequently borrowed from other models, such as VGGNet,^19^ ResNet,^20^ or DenseNet.^21^ In this study, we employed UNet and LinkNet with DenseNet backbone pretrained on ImageNet.^22^

We modified the classification head to incorporate a softmax activation with 3 output classes. The model was fine-tuned with a maximum learning rate of 0.0001 with the Adam optimizer and leaving other Adam parameters at the default settings.

We extended the model input by adding a Conv2D layer to learn 3 filters with kernel size (1,1), thus converting our single-channel grayscale input images to the 3 channels expected by the DenseNet backbone. Our loss function was categorical cross-entropy.

The models were fine-tuned on the raw single-class images and synthetic 2-class images, and the combinations of those. Random image augmentations were used to increase the generalizability of the model and included horizontal flip, vertical flip, and rotation of up to 45° followed by cropping to remove blank regions at the corners of the rotated images. The rotate-and-crop augmentation also introduced a small amount of feature scaling (zoom in) to the center of the image.

The training was performed on servers with NVIDIA CUDA 11.2 and Tesla P100-PCIE-16GB GPUs with parallelization.^23^ The models were fine-tuned and evaluated on python 3.6.9 with tensorflow 2.3.1, keras 2.4.0, and used the segmentation_models 1.0.1 library. Training used a batch size of 32 images.

## Results

All models were trained for 2400 epochs. Training on the single-class images required 20 hours, synthetic 2-class images required 30 hours, and training with both datasets required 51 hours.

### Quantitative Evaluation

Each of the trained models was evaluated on the single-class and synthetic 2-class reserved test images separately. Averaging of the Dice scores enabled us to perform an initial ranking of model performance (Table 2). We found that the UNet model performed slightly better when scored on the single class images (averaged Dice 0.9853), whereas the LinkNet model performed slightly better on the synthetic 2-class images (averaged Dice 0.9638). Our synthetic 2-class images introduce variations that do not present in the original dataset. This increased diversity can expose the model to a wider range of scenarios and improve its ability to handle more complicated cases (e.g., superficial/deep plexus present in a single image).

**Table 2:** Dice Scores on Reserved Test Images for 2 Model Architectures

Training Datasets	Model	Dice, Single-Class Test Image				Dice, Synthetic 2-Class Test Image				Combined (Simple) Average
		Superficial	Deep	Avas.	Avg.	Superficial	Deep	Avas.	Avg.	
Single-class	UNet	**0.9995**	0.8669	0.8953	0.9206	0.8875	0.6341	0.6410	0.7209	0.8208
Synthetic 2-class		0.9960	0.9708	0.9751	0.9807	0.9766	0.9545	0.9348	0.9553	0.9680
Single + synthetic 2-class		0.9920	**0.9788**	**0.9851**	**0.9853**	0.9715	0.9440	0.9209	0.9455	0.9654
Single-class	LinkNet	**0.9995**	0.9510	0.9558	0.9688	0.8824	0.7445	0.7194	0.7821	0.8755
Synthetic 2-class		0.9957	0.9380	0.9452	0.9596	0.9765	0.9488	0.9222	0.9492	0.9544
Single + synthetic 2-class		0.9958	0.9772	0.9816	0.9848	**0.9790**	**0.9635**	**0.9489**	**0.9638**	**0.9743**

Avas. = avascular; Avg. = average.

UNet with DenseNet backbone and LinkNet with DenseNet backbone. Training with combined single + synthetic 2-class data provided the best options for both models; LinkNet performed better overall.

Bold signifies the highest performance per column.

### Qualitative Evaluation

**Figure 3:**
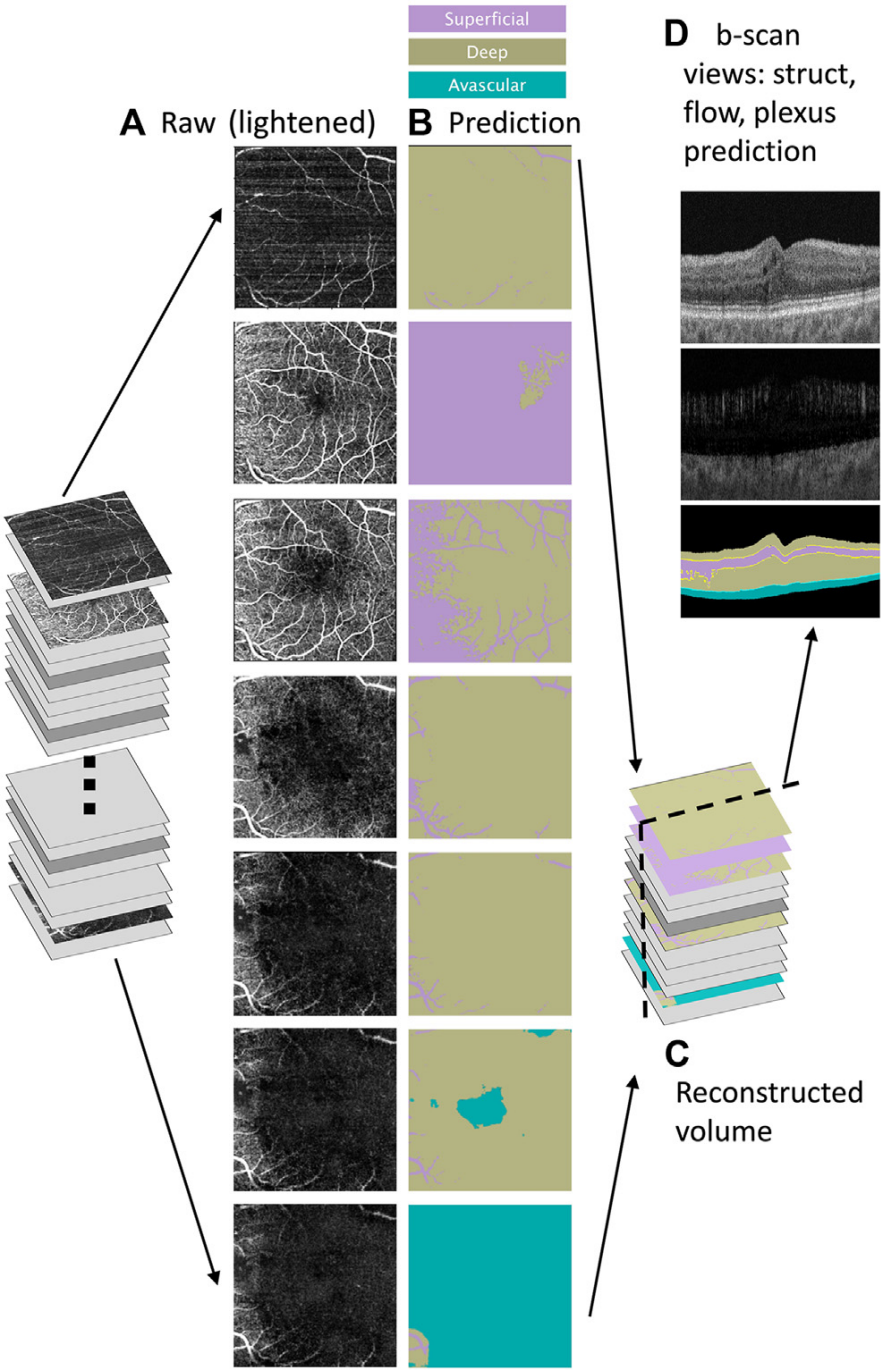
Thin slabs from an OCT angiography cube were split into (**A**) thin slabs (lightened somewhat for print), plexus predictions (**B**) on each thin slab were obtained from a trained model, these predictions were reassembled as a volume (**C**) and then sliced in the b-scan direction to perform qualitative analysis in comparison to flow and structure b-scans of the same b-scan slice (**D**).

**Figure 4:**
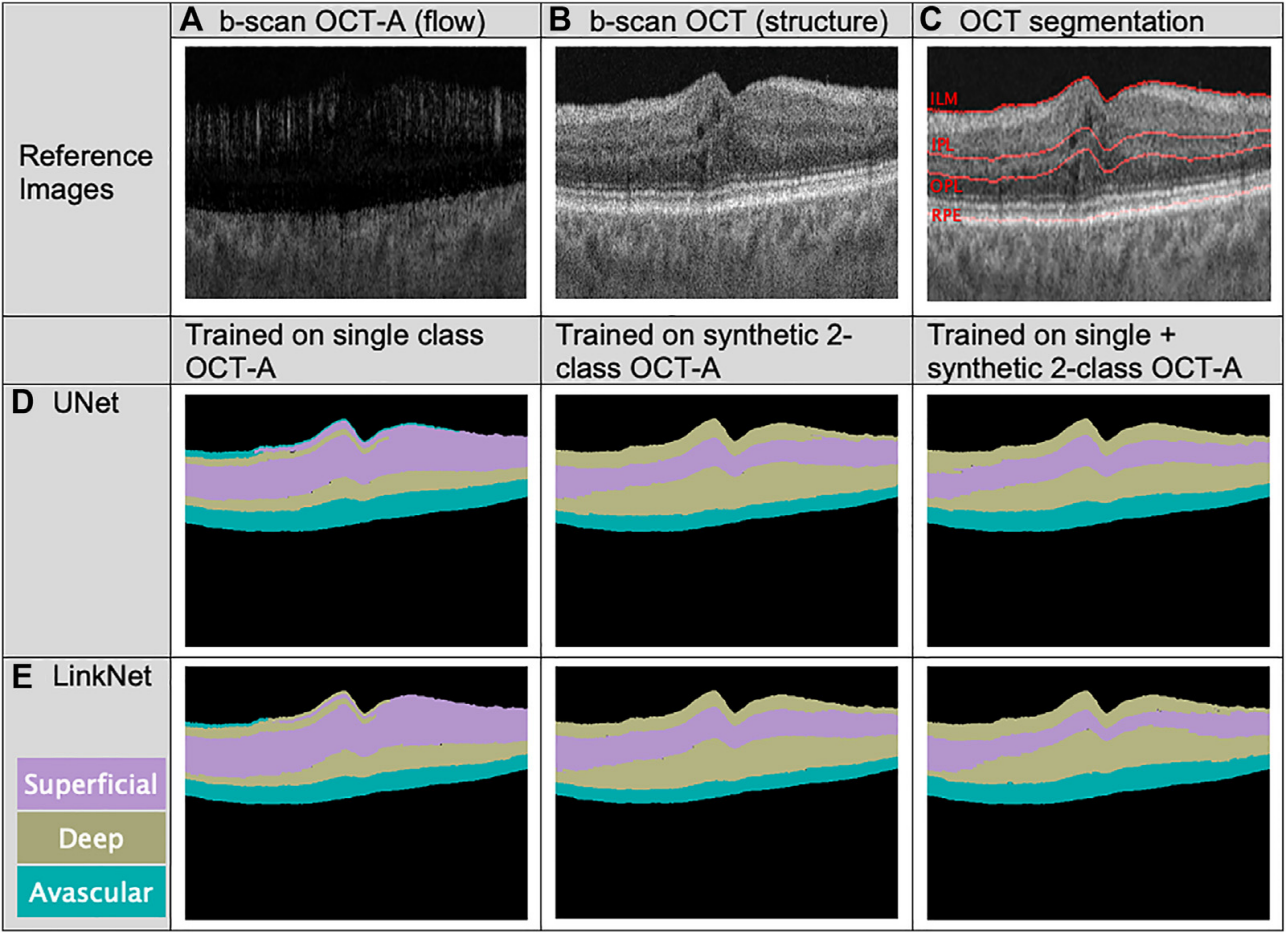
All images are from the same b-scan slice of a single OCT (OCTA) cube. Images (**A**) OCTA flow and (**B**) OCT structure and (**C**) OCT segmentation are for visual reference, with the boundaries provided by the layer segmentation algorithm shown for ILM, IPL, OPL, and RPE. Model predictions (**D**) UNet and (**E**) LinkNet correspond to the models that achieved the highest Dice scores as reported in Table 2. ILM = internal limiting membrane; IPL = inner plexiform layer; OCTA = OCT angiography; OPL = outer plexiform layer; RPE = retinal pigment epithelium.

For this evaluation, 500 × 500-pixel en face thin slabs were extracted from the OCTA cube as described earlier, downsampled to 256 × 256 pixels, and the models were run on the thin slabs (Fig 3A) to acquire the plexus predictions (Fig 3B). The predicted masks were then upsampled in a postprocessing step to create 500 × 500-pixel masks. Note that we did not explicitly provide individual vessel or plexus regions in our ground-truth, but instead created a blended image by generating an ovoid region with blurred edges. Nevertheless, the prediction masks often exhibited vascular plexus structure in their segmentation boundaries, which implies our model captures important/relevant patterns in the data. These prediction masks were mapped back onto the original OCTA thin slab regions throughout the volume to create a prediction cube (Fig 3C); for voxels included in more than one thin slab, the most likely class based on the combined predictions was assigned. The prediction cube was vertically sliced to observe the prediction as a b-scan view (Fig 3D), and the predictions outside the region of interest (e.g., in the vitreous region) were cropped from the final plots. Reference images of the OCT and OCT-A b-scans of a representative patient (Fig 4) enable side-by-side qualitative comparison of the predictions made by the top-performing models (Table 2).

**Figure 5:**
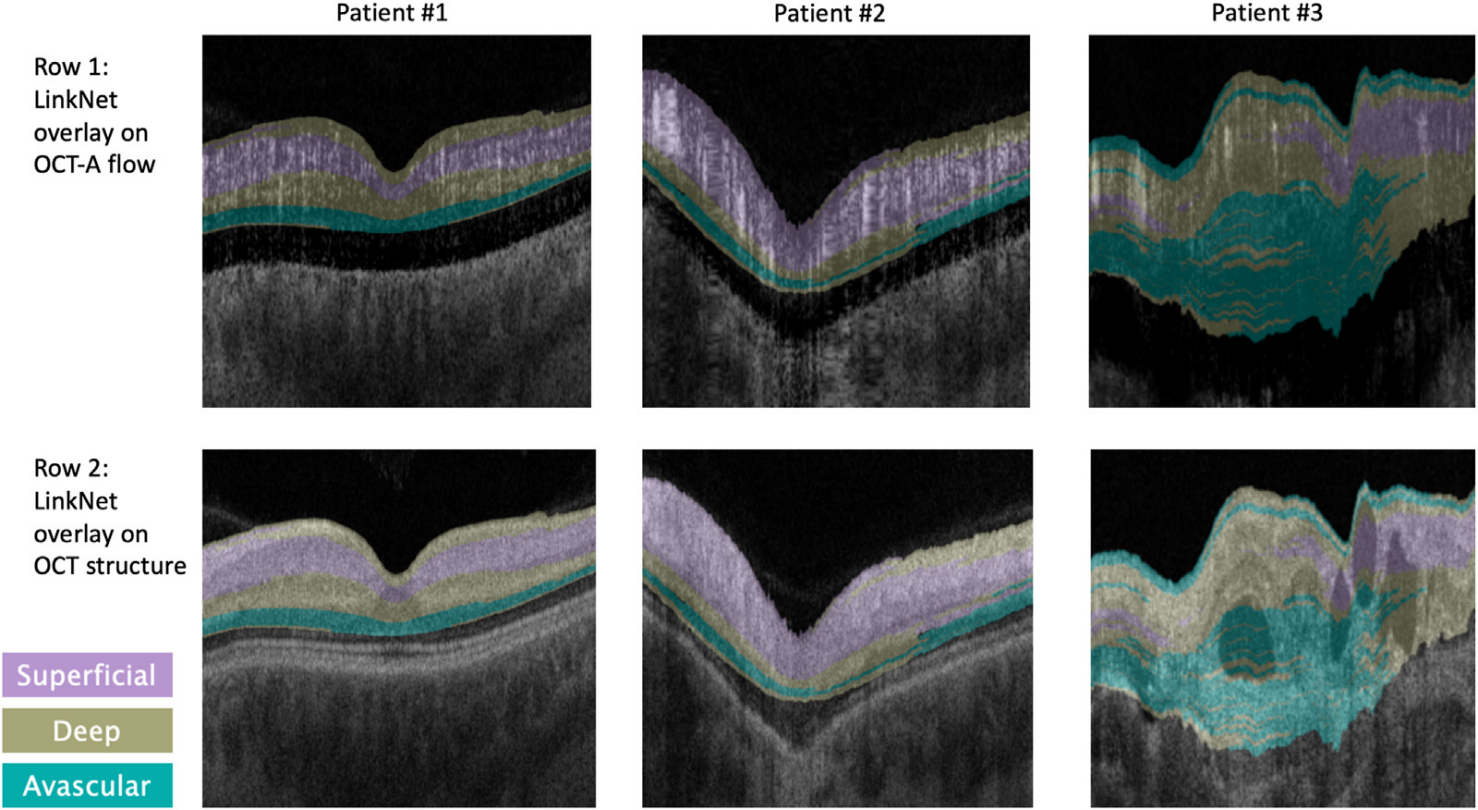
LinkNet predictions overlaid on flow images (top row) and OCT structure (bottom row) for 3 OCT angiography volumes from the reserved test set. The b-scans for patients #1 and #3 are through the center of the volume; the b-scan for patient #2 is a peripheral b-scan. OCTA = OCT angiography.

Predictions were made on en face thin slabs of OCTA cubes from the reserved test set and reassembled into prediction cubes. For 3 interesting cases, b-scan slices were taken from the unlabeled OCTA cube (flow) and from the OCT cube (structural) to serve as references for qualitative comparison, and the predictions from the top-performing LinkNet model were overlaid on these reference images (Fig 5).

## Discussion

In this manuscript, we present a method to train a deep learning model in the absence of structural OCT data to segment the superficial vascular plexus (SVP), deep vascular plexus (DVP), and avascular layer of the retina using only OCTA as input. With 235 OCTA cubes from 33 patients for training, test and validation, we achieved a >0.95 Dice score on a multiclass segmentation task using a mixed training set of labeled and synthetic data from handcrafted ground-truth masks. This model is one of the first fully automated plexus segmentation algorithms that only uses OCTA imaging as input.

Imaging studies have shown that the retinal plexuses do not travel consistently through all the retinal layers,^13^ and previously used rules of defining plexus boundaries from structural OCT segmentation boundaries are likely to be inconsistent throughout the whole retina if they follow absolute rules regarding plexus definition in relation to retinal layers. This may partially explain the finding of Spaide and Curcio,^24^ which showed that OCT instrument embedded plexus segmentation models in healthy eyes are inconsistent with their quality of segmentation especially near the center of the eye (fovea), where the plexuses start to merge on top of each other. One potential explanation for the decrease in performance is that all previous published plexus segmentation algorithms relied on structural OCT segmentations to infer retinal plexus boundaries^16^^,^^25^, ^26^, ^27^; if a patient has specific retinal pathology that affects the segmentation of the retinal layers on structural OCT, this will impede any of the aforementioned plexus segmentation models that use structural OCT. For patient 2 in Figure 5, the inferred B-scan is located at the peripheral retina. In this location, the retinal layers are poorly defined; thus, any automatic segmentation of retinal layers on structural OCT would be less accurate. Qualitatively, however, our network is able to identify the avascular, deep, and superficial layers of the vasculature. This same behavior is seen in Patient 3 of Figure 5, where there is significant retinal pathology. In this case, automated structural segmentation of the retinal layers would be severely affected, and inferring OCTA boundaries from the structural OCT is challenging. In this manuscript, our method is able to grossly assign plexus layers in the correct anatomical hierarchy even in significantly diseased retinas. Although further research is required to evaluate the model's performance on diseased eyes, these findings suggest that OCTA signal may be preserved in retinal disease that is independent of the structural layers, thus allowing for plexus segmentation without adequate structural OCT layer segmentation.

Qualitative evaluation of the segmentation outputs show that the model's performance varies depending on the data it was trained on. The models achieved the highest Dice score and best subjective qualitative evaluation when trained on the combined single-class and synthetic 2-class image dataset. As seen in Figure 5, the model predicts a layer of DVP superficial to the SVP. Initially this was thought to be mislabeling that could be postprocessed from the final prediction maps. However, after superimposing the structural OCT B-scan with the OCTA plexus segmentation mask, the misclassified DVP layer lies over the retinal nerve fiber layer (RNFL) on OCT. Previous anatomical studies for the retina have suggested that there is an additional unique plexus that exists as a subdivision of the SVP that is located within the RNFL; this has been referred to as the nerve fiber layer plexus, ganglion cell layer plexus, and radial peripapillary capillary plexus.^12^^,^^13^ It may be possible that the model's mislabeling of a superficial DVP layer on the most superficial layer of the retina will enable identification of this subdivision of the SVP.

Deep learning models are designed to perform their task according to the labeled data that are inputted. In this manuscript, we chose to use the SVP and the DVP because they represent the 2 larger networks on which there is general consensus. The intermediate capillary plexus, nerve fiber layer plexus, ganglion cell layer plexus, and radial peripapillary capillary plexus are smaller subdivisions of capillary networks described previously; however, authors have disagreement on the nomenclature and boundaries.^12^ We elected to not subdivide the SVP and DVP into these subdivisions because of the lack of consensus on their definitions and locations. Additionally, we elected not to segment the foveal avascular zone (FAZ) in the manually curated ground truth labels because it is extremely difficult to identify the boundary of where this begins in OCTA. In future work, a model could be trained with additional plexus layers and FAZ labels and likely achieve similar results, as this manuscript is a proof-of-concept that OCTA alone is sufficient for segmenting retinal plexus layers. An additional future application is fine-tuning the layer segmentation in an OCTA volume after rough boundaries have been defined using more conventional methods. For example, when determining the boundary between the superficial and deep capillary plexuses, the existence of larger vessels in the superficial vasculature may produce local deformations of this boundary that may be difficult to follow. Finally, this technique could be used in conjunction with existing OCTA vessel segmentation techniques to identify vessels with their corresponding plexus, and these plexus segmentations may aid in studying retinal microvascular changes across a wide range of diseases.

### Limitations

Although the model has achieved high Dice scores (>0.95), the qualitative comparison with b-scans highlights remaining challenges, including whether to handle the large vessel tails (artifacts) prior to training, within the model, or in a postprocessing step. In the current images, the presence of the large vessel in the thin slabs deeper in the OCTA cube is extending the superficial plexus somewhat deeper than expected. Additionally, there are segmentation errors in eyes with more severe pathology. This may be improved by supplementing the training data with more diseased eyes.

Generation of thin slabs that overlap to thin slabs above and/or below by a few pixels can lead to a better solution through multiple evaluations of different instances surrounding the data; at the same time, the generation of these thin slabs may be hit-or-miss and limit the ability of the algorithm to learn from other sets of data or to generalize in some cases of pathology.

Recent work^12^ suggests that 4 distinct plexuses may be identified, including the intermediate capillary plexus and a plexus lying along the RNFL. Our work used a simplified method with only weak labels of the superficial and deep plexuses; this is due in part to the difficulty in consistently distinguishing more than 2 plexuses in current OCTA data by automated methods. The second reason is that these plexuses are not easy (or are impossible) to perfectly separate by slabs because they do not spread perfectly horizontally and the retina is very thin.

The simplified en face datasets we used did not identify the FAZ and therefore trained the model to include this region with the superficial or deep plexus. The FAZ is not found in all patients, and although it was likely identifiable in our dataset, for this work we elected to explore the progress that could be made using the overly simplified classes and weak labels without delineating the FAZ. As a result, our model does not commit the error of assuming layers are of uniform thickness near the fovea,^24^ although neither does it identify an avascular region for the FAZ. This may be the subject of future work.

In this work, we trained a deep learning model that is able to segment the superficial plexus, deep plexus, and avascular layers of the retina using only OCTA as input. By augmenting our training set with synthetically generated 2-class images from adjacent layer classes, we were able to improve the model's performance without requiring multiclass manually segmented images. Our method demonstrates that OCTA alone has adequate signal to identify the plexus layers and provides a technique that could be used if segmented structural OCT data are unavailable or segmentation is unreliable due to retinal disease.

## Data Availability

The process for requesting and receiving data are changing and we are unable to ensure the availability at this time.
